# Is Bromide Potass. and Sulph. Morphia an Antidote for Poisoning by Strychnine?

**Published:** 1877-10

**Authors:** J. T. Moore

**Affiliations:** Cairo, Ga.


### IS BROMIDE POTASS. AND SULPH. MORPHIA AN ANTIDOTE FOR POISONING BY STRYCHNINE?

By J. T. MOORE, M.D., Cairo, Ga.

I was called to a case of strychnine poison a few years ago, in the person of Mr. F., a very prominent citizen, living near the dividing line between Georgia and Florida.

He had for several years been subject to attacks of cramp colic, and had been advised by his physician to take morphine to relieve the pains, at the time of which I am writing. He had a very severe attack at night, and called for the morphine. His wife, in her haste and fright, gave him strychnine, and he did not discover the mistake until on rising from bed and attempting to go to the hearth to vomit, (the morphine having nauseated his stomach,) his legs gave way under him—in other words, were paralyzed. The strychnine had been taken at four o'clock, A.M., and I was called at six, A.M. I found him in all the agonies of mind and body that strychnine could produce. His greeting to me was: "Doctor, I have but a few minutes to live." I began treatment by giving:

℞. Brom. potass . . . . . ʒj

Sulph. morphia, . . . . gr. ss M.

This I repeated every half hour until I had given two grains of morphine. I then diminished the dose and increased the interval to an hour, giving about one-eighth of a grain of morphine to eight or ten grains of potass. By one o'clock, P.M., I thought him safe from the strychnine, but fearing gastric derangement, I gave him the following:

℞.—Calomel, . . . . . . gr. xii.
Pulv. Dover,
Pulv. Ipecac, aa. . . . . . gr. vj.
M. Divide in vi. powders.

Sig.—one every three hours. Give castor oil four hours after the last powder has been taken.

I called to see him the following morning, and found him with some fever. The medicine had not acted, and I continued the above prescription. At twelve o'clock he had a very large action, passing a large quantity of grape seed, and the fever began subsiding immediately. He passed a comfortable night, resting well. In the morning he was entirely clear of fever, and was dismissed.

I did not give an emetic in the above case, because I thought the poison had been taken up by the absorbents and distributed through the system. The paralysis of the limbs furnished sufficient evidence of the fact, and, consequently, I thought it would be valuable time lost to attempt to produce emesis.

